# Association between hypertension and neurovascular inflammation in both normal-appearing white matter and white matter hyperintensities

**DOI:** 10.1186/s40478-022-01497-3

**Published:** 2023-01-04

**Authors:** Gemma Solé-Guardia, Emma Custers, Arthur de Lange, Elyne Clijncke, Bram Geenen, Jose Gutierrez, Benno Küsters, Jurgen A. H. R. Claassen, Frank-Erik de Leeuw, Maximilian Wiesmann, Amanda J. Kiliaan

**Affiliations:** 1 Department of Medical Imaging, Anatomy, Radboud University Medical Center, Donders Institute for Brain, Cognition and Behavior, Center for Medical Neuroscience, Preclinical Imaging Center PRIME, Radboud Alzheimer Center, Nijmegen, The Netherlands; 2 Department of Neurology, Vagelos College of Physicians and Surgeons, Columbia University Medical Center, New York, NY USA; 3 Department of Pathology, Radboud University Medical Center, Nijmegen, The Netherlands; 4 Department of Geriatrics, Radboud University Medical Center, Donders Institute for Brain, Cognition and Behavior, Center for Medical Neuroscience, Radboud Alzheimer Center, Nijmegen, The Netherlands; 5 Department of Neurology, Radboud University Medical Center, Donders Institute for Brain, Cognition and Behavior, Center for Medical Neuroscience, Nijmegen, The Netherlands

**Keywords:** Small vessel disease, Hypertension, White matter hyperintensities, Normal-appearing white matter, *Post-mortem* MRI, Inflammation

## Abstract

The major vascular cause of dementia is cerebral small vessel disease (SVD), including white matter hyperintensities (WMH) amongst others. While the underlying pathology of SVD remains unclear, chronic hypertension and neuroinflammation are recognized as important risk factors for SVD and for the conversion of normal-appearing white matter (NAWM) to WMH. Unfortunately, most studies investigating the role of neuroinflammation in WMH relied on peripheral blood markers, e.g., markers for systemic or vascular inflammation, as a proxy for inflammation in the brain itself. However, it is unknown whether such markers accurately capture inflammatory changes within the cerebral white matter. Therefore, we aimed to comprehensively investigate the impact of hypertension on perivascular- and neuroinflammation in both WMH and NAWM. We conducted high field brain magnetic resonance imaging (MRI), followed by (immuno-)histopathological staining of neuroinflammatory markers on human *post-mortem* brains of elderly people with a history of hypertension (n = 17) and age-matched normotensive individuals (n = 5). MRI images were co-registered to (immuno-)histopathological data including stainings for microglia and astroglia to assess changes in MRI-based WMH at microscopic resolution. Perivascular inflammation was carefully assessed based on the severity of perivascular astrogliosis of the smallest vessels throughout white matter regions. Hypertension was associated with a larger inflammatory response in both WMH and NAWM. Notably, the presence of close-range perivascular inflammation was twice as common among those with hypertension than in controls both in WMH and NAWM, suggesting that neurovascular inflammation is critical in the etiology of WMH. Moreover, a higher degree of microglial activation was related to a higher burden of WMH. Our results indicate that neuro(vascular)inflammation at the level of the brain itself is involved in the etiology of WMH. Future therapeutic strategies focusing on multitarget interventions including antihypertensive treatment as well as neuroinflammation may ameliorate WMH progression.

**Supplementary Information:**

The online version contains supplementary material available at 10.1186/s40478-022-01497-3.

## Introduction

Cerebral small vessel disease (SVD) causes about 20% of all strokes and is a major etiological factor in dementia worldwide [26, 30]. As it remains difficult to visualize the smallest vessels in vivo in human brains, the identification of SVD relies on magnetic resonance imaging (MRI) markers [32], including white matter hyperintensities (WMH). WMH, but also areas at risk for WMH (so called normal-appearing white matter (NAWM) on fluid-attenuated inversion recovery (FLAIR) MRI [33]), are strongly associated with common cardiovascular risk factors, including hypertension [1, 8, 30]. Hypertension has been proposed to trigger both low grade systemic but also vascular inflammation and microglial activation in the brain (neuroinflammation) [5]. Neuroinflammation—the inflammatory response within the central nervous system (CNS) [21]—is increasingly recognized as an early event in the pathogenesis of SVD [18] and as such may be involved in the conversion of NAWM to WMH. Therefore a better knowledge on the association between hypertension and both neuro- and vascular inflammation may help to better understand the pathogenesis of WMH.

However, previous studies investigated the relation between SVD and both neuro- and vascular inflammation only at the level of peripheral blood markers (reviewed by [18]), rather than at the actual site of the presumed CNS inflammation. These approaches may therefore not have completely captured neuro(vascular)inflammatory changes within the CNS. Novel evidence from positron emission tomography (PET) imaging has indeed shown a strong association between microglial activation and SVD, especially in patients with hypertension [17]. Unfortunately, the low spatial resolution of PET makes it difficult to anatomically correlate these lesions to MRI markers of SVD. Consequently, the extent of neuro(vascular)inflammation in WMH and NAWM, remains unclear.

Therefore, we aimed to investigate the relation between hypertension and both neuro- and perivascular inflammation in WMH and NAWM. First, we used (immuno-)histopathological evaluation of neuroinflammation in WMH and NAWM as assessed by human *post-mortem* high field (HF) 7 Tesla MRI from individuals with hypertension and compared that with normotensive individuals. Additionally, we analyzed cerebrovascular inflammation based on the severity of astrogliosis surrounding small vessels. Finally, we examined the effect of the degree of WMH burden on both neuro- and perivascular inflammation.

## Materials and methods

### Cases

Our study cohort consists of twenty-two *post-mortem* human brains that were included through the body donors’ program at the Radboud university medical center, Nijmegen, The Netherlands between 2015 and 2019. Patients were included when hypertension (according to national guidelines at that time) was reported in their medical records. Age-matched individuals were included as controls when no record of hypertension or use of antihypertensive medication was identified in their medical reports. Exclusion criteria were the presence of a brain tumor and/or metastases, and/or territorial infarctions and/or atrial fibrillation (based on medical history or when identified during *post-mortem* MRI) as the latter two may also result in thromboembolic MRI lesions that mimic MRI markers of SVD. All individuals signed informed consent to use their medical records for research purposes, autopsy and use of tissue. The study was approved by the Medical Ethics Review Committee region Arnhem-Nijmegen (Commissie Mensgebonden Onderzoek (CMO) region Arnhem-Nijmegen file No. 2017–3941).

### Vascular risk factors in medical history

Presence of diabetes was based on either a reported diagnosis, or the use of antidiabetic medication in the medical records. Hypercholesterolemia was based on a history of statin use and/or report of hypercholesterolemia or elevated cholesterol levels in the medical records. Classification of vascular risk factors was done in accordance with national guidelines at the time of death. Smoking and/or alcohol use were reported as ever/never-smoking or drinking alcohol.

### Tissue processing

**Fig. 1:**
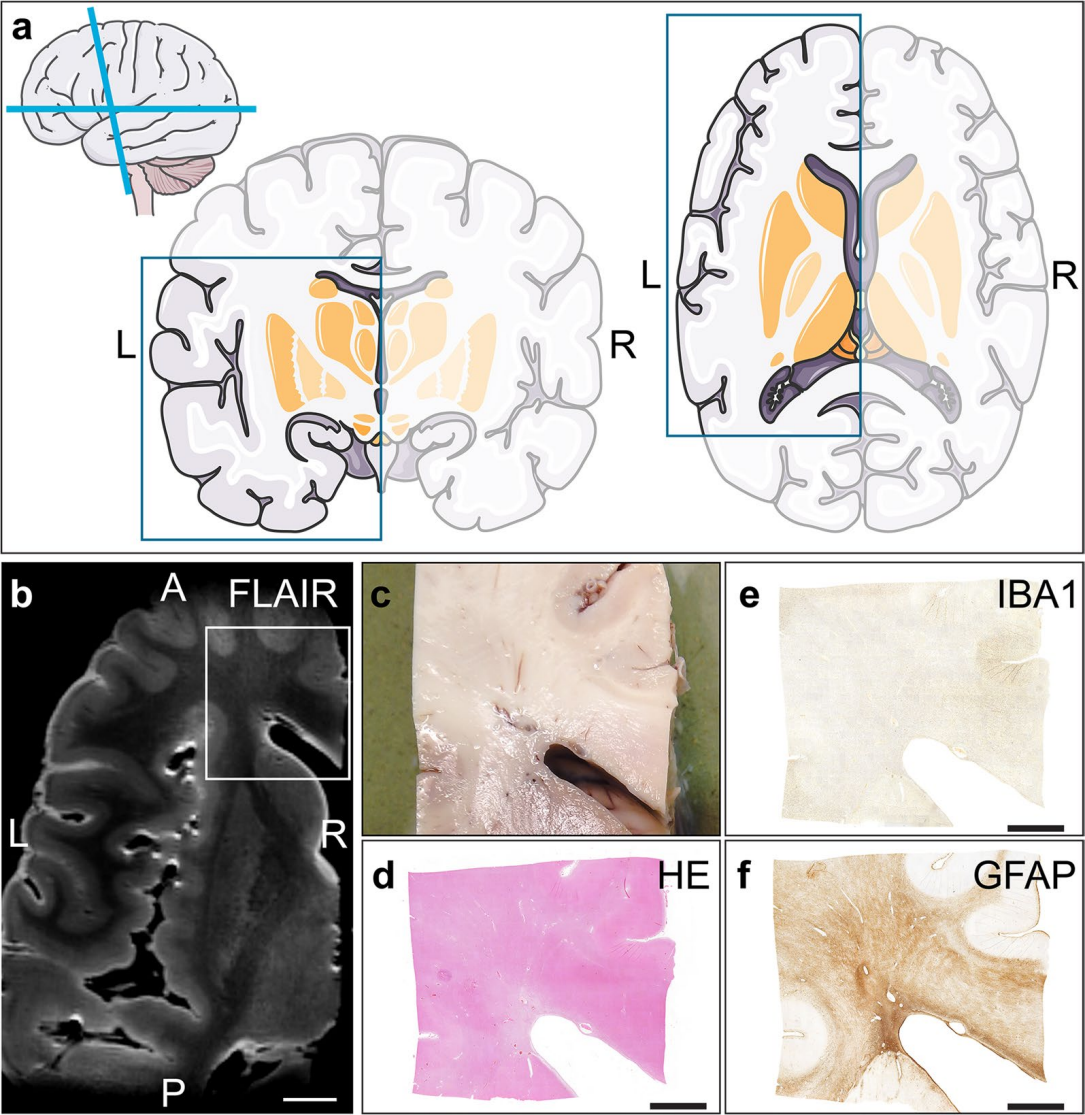
Tissue preparation. The left hemisphere was divided into dorsal and ventral part for HF (7 Tesla) MRI scanning. **a** Schematic brain images. The blue squares illustrate the dimensions of the ventral part of the left hemisphere. **b** Corresponding HF MRI fluid-attenuated inversion recovery (FLAIR) axial slab (white scale bar = 1 cm). The white rectangle illustrates the tissue blocks of approximately 2 × 2 × 0.5 cm taken from the (**c**) biopsy. (Immuno-)histopathology of (**d**) haematoxylin/eosin (HE), (**e**) ionized calcium-binding adapter molecule 1 (IBA1) to detect macrophages and microglia, and (**f**) glial fibrillary acidic protein (GFAP) to detect astrocytes. This figure was partly generated using “Neurology” images from Servier Medical Art (https://smart.servier.com), licensed under a Creative Commons Attribution 3.0 Unported License. (black scale bar = 0.5 cm) (*L* left, *R* Right, *A* Anterior, *P* Posterior)

After autopsy, brains were removed from the skull and fixed in ~ 8% formalin for at least 2 months before imaging. Prior to HF MRI scanning, the brainstem with cerebellum and the circle of Willis were removed. Because of space limitations of the HF coil, the brain was cut mid-sagittal and the left hemisphere was horizontally divided into a dorsal and a ventral part using refence landmarks like the corpus callosum to keep tissue processing comparable between individuals. These parts were subjected to HF 7 Tesla MRI to visualize radiological markers of SVD. After careful examination of HF MRI data, the ventral part of each brain was divided into a slab of approximately 10 cm length along the horizontal plane (Fig. 1). For (immuno-)histopathology these slabs were cut along the coronal plane into blocks of approximately 2 × 2 × 0.5 cm containing periventricular white matter.

### Post-mortem MRI

Before scanning, the specimens were removed from the formalin solution and washed in tap water for at least 24 h. Next, the ventral part of the left hemisphere was placed in a plastic bag filled with Fluorinert (3 M, FC-3283, Maplewood, MN, USA), a proton-free liquid. Air bubbles were removed by hand and by ultrasonic bath (Bransonic 221, Danbury, CT, USA). Specimens were scanned at room temperature in a Bruker 7 Tesla Clinscan MRI system (Bruker Biospin, Ettlingen, Germany) interfaced with a Siemens Syngo VB15 console. Scans were acquired with a T1-weighted sequence at a resolution of 400 × 400 × 400 µm (repetition time (TR) = 20 ms, echo time (TE) = 1 ms, 1 average); T2*-weighted sequence at a resolution of 400 × 400 × 400 µm (TR = 20 ms, TE = 13 ms, 1 average); T2-weighted sequence at a resolution of 400 × 400 × 400 µm (TR = 10,780 ms, TE = 25 ms, 2 averages); FLAIR sequence at a resolution of 500 × 500 × 500 µm (TR = 8200 ms, TE = 39 ms, 2 averages).

*Post-mortem* MRI scans were acquired with a mean of 11 months after death (standard deviation (SD) 11.9 months), MRI acquisition time was not different between individuals with hypertension and age-matched controls (*p* = 0.756). All acquired MRI images were displayed using MANGO (version 4.1; Multi-image Analysis GUI); Research Imaging Institute, University of Texas health science center, TX, USA; www.ric.uthscsa.edu/mango) [15]. Periventricular WMH severity was visually evaluated on MRI FLAIR sequence following the Fazekas scoring system [6]. Volumetric segmentation of periventricular WMH within the ventral part of the left hemisphere was performed semi-automatically through ITK-SNAP (version 3.8.0; www.itksnap.org) [34]. All MRI scans were evaluated by three blinded experienced raters (GSG, MW, FEdL).

### (Immuno-)histochemistry

Tissue blocks were embedded in paraffin and sectioned at 4 µm thickness. These sections were stained with haematoxylin/eosin (HE) following standard histology protocols. (Immuno-)histochemistry was performed on adjacent sections for ionized calcium-binding adapter molecule 1 to detect macrophages and microglia (IBA1; rabbit, ab178846; Abcam, Cambridge, UK; 1:2000, RRID: AB_2636859) and glial fibrillary acidic protein to detect astrocytes (GFAP; rabbit, Z0334; Dako, Santa Clara, CA, USA; 1:600, RRID: AB_10013382).

Sections were first deparaffinized in xylene, rinsed through graded ethanol series and ultimately in demi water. For all immunostainings, sections were processed further using a fully automated immunostainer (Lab Vision Autostainer 360; Thermo Fisher Scientific) and the EnVision FLEX visualization system (K8000, Agilent, RRID: AB_2890017), according to manufacturer’s instructions. Briefly: sections were rinsed in EnVision FLEX Wash Buffer (K800721-2; Agilent, Santa Clara, CA, USA) for 5 min, followed by 5 min in Peroxidase-Blocking Reagent, and a 5-min rinse in EnVision FLEX Wash Buffer. Sections were incubated with the aforementioned primary antibody for 60 min. After incubation, sections were rinsed for 10 min in EnVision FLEX Wash Buffer and incubated for 15 min with EnVision FLEX + rabbit (LINKER) (K800921; Agilent, Santa Clara, CA, USA). After another 10-min rinse in EnVision FLEX Wash Buffer, sections were incubated with EnVision FLEX HRP Solution (Agilent, Santa Clara, CA, USA) for 30 min, then another 10-min rinse in EnVision FLEX Wash Buffer. Sections were incubated with a mixture of EnVision FLEX 3,3'-diaminobenzidine (DAB) + and Substrate Solution (Agilent, Santa Clara, CA, USA) for 10 min and rinsed in tap water for 10 min. GFAP sections were counterstained using hematoxylin before dehydration in ethanol and xylene, and cover slipping.

### MRI-pathology co-registration and Regions of interest (ROIs)

Prior to the registration of the MRI images to the HE stained sections, the MRI data of the 22 individuals was loaded into MANGO (Research Imaging Institute, University of Texas health science center, TX, USA; www.ric.uthscsa.edu/mango) [15]. The MRI data (T1-weighted, T2-weighted, T2*-weighted, FLAIR) was compared to the HE reference section to select the corresponding 2D MRI slide. The selection of 2D MRI slides was based on the comparison of different anatomical landmarks (i.e., corpus callosum, caudate nucleus, cortex) across MRI-pathology performed by experienced neuroanatomists (GSG, BG). Then MRI data was registered to the HE reference section based on manual landmark selection using a custom MATLAB script (MATLAB R2020a; MathWorks Inc., Natick, MA, USA). Briefly, MRI data was extracted to previously selected 2D axial slices. At least 10 landmarks were selected on both 2D MRI slide and HE reference section. Next, the MRI 2D images were warped [20] and cropped based on the HE reference.

After MRI-pathology registration, NAWM and WMH were manually segmented based on MRI by different experienced assessors who were blinded to presence of hypertension, sex and other risk factors for white matter lesions. The different regions of interest (ROIs) corresponding to NAWM and WMH were defined for further analysis when agreement was met for at least 2 assessors. MRI and (immuno-)histochemistry data from each individual was subsequently clustered into these ROIs for statistical analysis.

### Post-processing of sections

All stained sections were digitized on a Pannoramic 1000 slide scanner (3DHISTECH Ltd, Hungary) employing a 20× magnifying objective. All high-resolution digital images (0.25 µm/pixel) were both visualized and exported to tag image file format (TIFF) (1:4 scale, 8-bit, jpeg with 80% compression) using CaseViewer software (version 2.4; 3DHISTECH Ltd, Hungary). Per specimen, all available stainings were co-registered to corresponding HE through a custom written intensity-based automated multimodal registration MATLAB script (MATLAB R2020a; MathWorks Inc., Natick, MA, USA) [19]. Prior to the co-registration, the images were cropped based on automatic tissue detection. Next, the sections stained for either GFAP or IBA1 were registered to the HE reference section. The result of the registration was visualized via an overlay montage of the HE reference section with IBA1 or GFAP. When the automated registration script failed to accurately register (other) (immuno-)histopathology to HE, these were manually registered based on landmark selection in the target-stained section and HE reference section. 1/22 specimen could not be co-registered to its respective IBA1 staining due to imaging limitations.

### Detection and quantification of (perivascular) inflammation

After this multimodal registration, stained sections containing periventricular white matter from all individuals were computationally segmented in ImageJ-MATLAB (version 1.53c, National Institute of Health, Bethesda, MD, United States; MATLAB R2020a; MathWorks Inc., Natick, MA, USA) [9] (we used the color deconvolution tool [25] for GFAP). Analyses were performed on aforementioned MRI-manually segmented ROIs (WMH, NAWM) for each individual. Intensity threshold was used to isolate the target staining from background. To account for varying staining intensities across individuals, the intensity threshold was determined by examining mean intensity values of positive stained cells within ROIs across all individuals for IBA1 and GFAP, separately. Threshold settings based on the overall mean intensity were checked on individual basis. IBA1 intensity was used as a marker for microglial activation [10], since it corresponds to increased IBA1 within microglial cells. Stained area was calculated for IBA1 and GFAP. Similarly, GFAP intensity was used as a marker for astrocytic activation [12]. Frequency of positive stained microglial cells (IBA1) was automatically counted by ImageJ (number per mm^2^). As GFAP-positive astroglia were often found forming scars in WMH, we did not include astroglia count. In our analysis, we also investigated whether microglial cells (IBA1) showed morphological changes. Based on previous research, it is known that resting microglial cells show a ramified morphology that upon activation changes towards an amoeboid/round shape with shorter ramifications [2]. Therefore, circularity (range 0 to 1) was calculated for IBA1-positive microglial cells; higher circularity values correlate to a rounder/more amoeboid morphology, which is indicative of an activated inflammatory state. Additionally, we measured average cellular length by ImageJ Measure Skeleton Length tool [23] to assess changes in microglial ramifications.

In addition to using GFAP staining to assess neuroinflammation in the brain parenchyma, we used GFAP staining to study perivascular inflammation. Perivascular inflammation was studied on astroglia adjacent to blood vessels within 15 µm. Particularly, perivascular inflammation was evaluated based on the following criteria using the range of perivascular inflammation observed across all individuals: grade 0 (none), grade 1 (mild; < 100 perivascular astroglia per mm^2^), grade 2 (moderate; 100–300 perivascular astroglia per mm^2^) and grade 3 (severe; > 300 perivascular astroglia per mm^2^ or presence of astrogliotic scar surrounding the blood vessels). Additionally, for each vessel presence of close-range perivascular inflammation was assessed. We examined perivascular inflammation within uniformly predefined regions in NAWM and WMH to avoid misclassification. After examination, an average was computed for mild, moderate, severe perivascular- and close-range perivascular inflammation per ROI for all individuals. The number of vessels graded did not differ between groups (*p* = 0.501) nor regions (*p* = 0.094).

### Statistics

Means and SD were calculated for all continuous variables, as well as frequencies and percentages for categorical variables. When assumptions on normality and homogeneity were not met, we used a natural log transformation. We used multivariate analysis of variance (ANOVA) for group comparisons of age, *post-mortem* delay, body mass index (BMI) and WMH volume. Relationships between categorical variables were explored using a Chi-square (χ^2^).

Means for neuroinflammatory markers and perivascular neuroinflammation (individuals with hypertension vs. controls, WMH vs. NAWM) were analyzed using ANOVA, controlled for age, sex and fixation-(immuno-)histochemistry interval, with a Bonferroni correction for multiple testing.

To examine the effect of the severity of WMH burden, we stratified individuals in two groups based on their Fazekas score (mild: Fazekas 0–1; moderate to severe: Fazekas 2–3) [6]. Neuroinflammatory markers and perivascular inflammation across WMH burden groups were analyzed using ANOVA, controlled for age, sex and fixation-(immuno-)histochemistry interval, with a Bonferroni correction for multiple testing.

Results were considered statistically significant when *P* ≤ 0.05. Statistical analysis of the data was performed using IBM SPSS statistics 25 SPSS (IBM Corporation, Armonk, NY, USA).

## Results

### Study population

Twenty-two individuals (full demographic information is provided in Table 1; see Additional file 1 for Table S2 including detailed cause of death). 17 with hypertension and 5 aged-matched individuals with no clinical record of hypertension, were included in this study. No differences were observed in demographics (age, gender distribution and *post-mortem* delay) and risk factors (apart from hypertension) between age-matched controls and individuals with hypertension. The group of individuals with hypertension had a greater WMH burden than the control group, both qualitatively (Fazekas score) (*p* = 0.02) (distribution in Table 1) and through volumetric quantification of WMH (*p* = 0.02).

**Table 1:** Demographic and clinical characteristics of the study cohort

	Total (n = 22)	Controls (n = 5)	Individuals with hypertension (n = 17)	*p value*
*Demographics*				
Age, mean ± SD, years	80.6 ± 8.1	80.2 ± 8.6	80.7 ± 8.2	*p* = 0.905
Sex, female, n (%)	10 (45.5%)	3 (60.0%)	7 (41.2%)	*p* = 0.457
*Post-mortem* delay, mean ± SD, hours	22.4 ± 6.8	20.0 ± 2.9	23.1 ± 7.4	*p* = 0.375
*Risk factors*				
BMI, mean ± SD, kg/m^2^	23.1 ± 4.4	23.8 ± 3.8	22.9 ± 4.7	*p* = 0.756^a^
Diabetes, n (%)	5 (22.7%)	1 (20.0%)	4 (23.5%)	*p* = 0.869
Hypercholesterolemia, n (%)	11 (50.0%)	1 (20.0%)	10 (58.8%)	*p* = 0.127
Smoking, n (%)	7 (31.8%)	2 (40.0%)	5 (29.4%)	*p* = 0.655
Alcohol use, n (%)	3 (13.6%)	0 (0%)	3 (17.6%)	*p* = 0.312
*WMH burden*				
Fazekas score, moderate to severe WMH (Score ≥ 2), n (%)	10 (45.5%)	0 (0%)	10 (58.8%)	*p* = 0.020*
WMH volume ^b^, mean ± SD, mL	1.40 ± 0.68	0.91 ± 0.26	1.54 ± 0.70	*p* = 0.020*
*Fazekas score*				
0, n (%)	3 (13.6%)	1 (20.0%)	2 (11.8%)	
1, n (%)	9 (40.9%)	4 (80.0%)	5 (29.4%)	
2, n (%)	9 (40.9%)	0 (0%)	9 (52.9%)	
3, n (%)	1 (4.6%)	0 (0%)	1 (5.9%)	
*Modified Fazekas score*				
Mild [0–1], n (%)	12 (54.5%)	5 (100%)	7 (41.2%)	
Moderate [2], n (%)	9 (40.9%)	0 (0%)	9 (52.9%)	
Severe [3], n (%)	1 (4.6%)	0 (0%)	1 (5.9%)	

*BMI* body mass index, *SD* standard deviation, *WMH* white matter hyperintensity

*p < 0.05

^a^Data missing for *n* = 6 for BMI

^b^These measures correspond to the WMH volume from the left hemisphere

### Neuroinflammation

#### Microglia

**Fig. 2:**
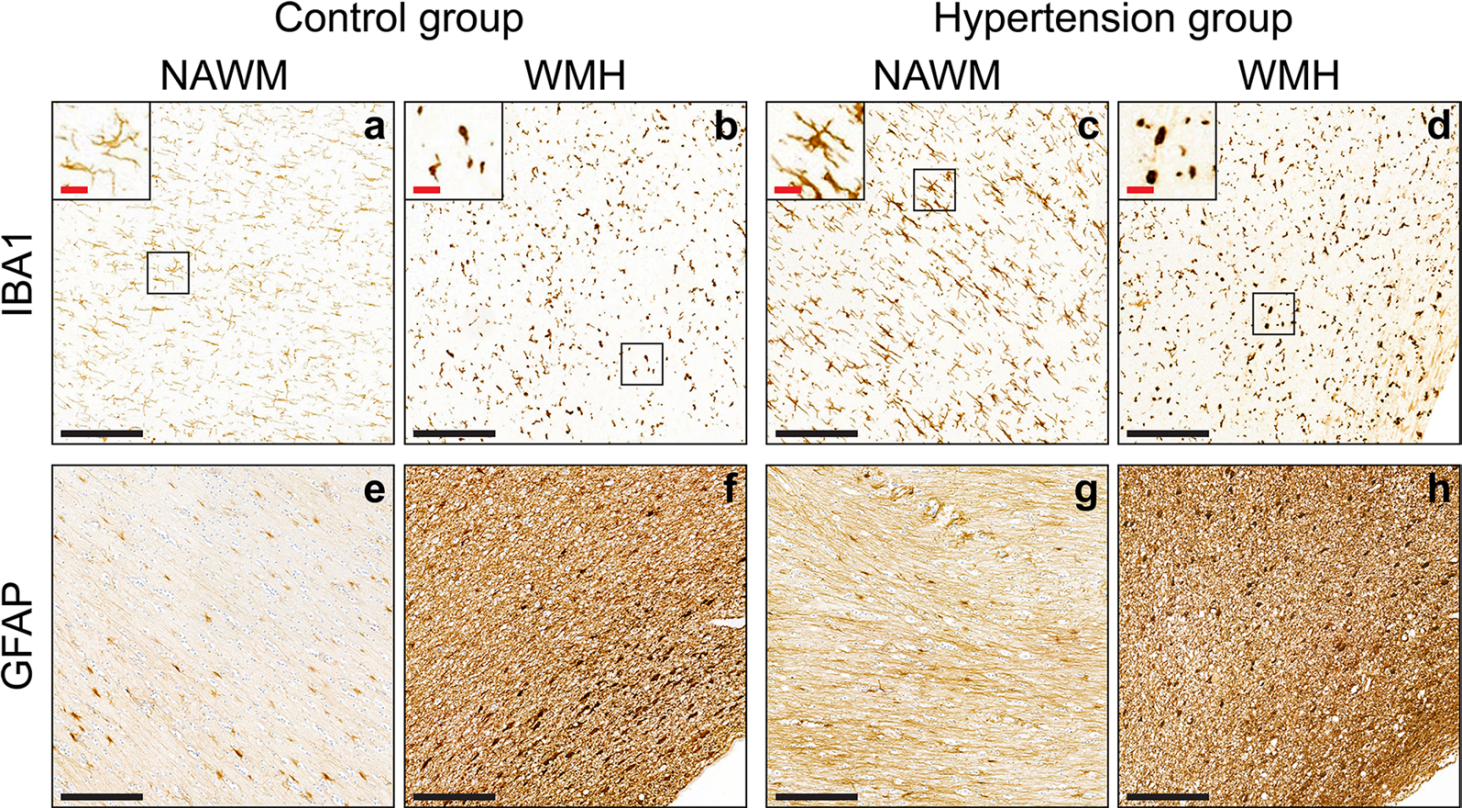
(Immuno-)histopathological characterization of inflammation of age-matched controls and individuals with hypertension within white matter. Representative images of ionized calcium-binding adaptor molecule 1 (IBA1) at X10 magnification of normotensive individuals (**a**, **b**) and individuals with hypertension (**c**, **d**). **a**, **c** correspond to normal-appearing white matter (NAWM) and **b**, **d** to white matter hyperintensity (WMH). The black boxes indicate regions of interest placed on the upper left corner of **a**–**d** at X20 magnification. Examination of amount of IBA1 showed more microglial activation (*p* = 0.01), slightly lower microglial area (*p* = 0.036), and a rounder/more ameboid cellular shape (*p* = 0.002) in individuals with hypertension (**c**, **d**) compared to controls (**a**, **b**). In WMH (**b**, **d**), we observed more microglial activation (*p* < 0.001), together with morphological changes in microglial cells. These changes were characterized by shortening of cellular ramifications (*p* = 0.014) and a rounder/more ameboid cellular shape (*p* < 0.001). Representative images of glial fibrillary acidic protein (GFAP) at ×10 magnification of normotensive (control) individuals (**e**, **f**) and individuals with hypertension (**g**, **h**). **e**, **g** Correspond to NAWM and **f**, **h** to WMH. Analysis of GFAP staining revealed that hypertension (**g**, **h**) leads to larger gliosis area (*p* = 0.005) compared to controls (**e**, **f**). Additionally, those individuals with hypertension showed an overall larger astrocytic activation (*p* < 0.001) than the control group. When comparing across white matter regions, WMH (**f**, **h**) showed larger GFAP positive area (*p* = 0.004) and astrocytic activation (*p* = 0.012) than NAWM (**e**,**g**). (black scale bar = 200 µm; red scale bar = 25 µm)

A higher microglial activation was detected in individuals with *hypertension* compared to controls in both WMH and NAWM (*p* = 0.010; Fig. 2 and Table 2). We observed that the morphology of the microglial cells of individuals with hypertension showed both increased circularity (amoeboid shape) (*p* = 0.002) and a slight decrease in microglia area (*p* = 0.036) in both WMH and NAWM compared to controls.

**Table 2:** Neurovascular inflammation by groups and regions of interest

	Control		Individuals with hypertension		*p* value^a^	
	NAWM mean ± SD	WMH mean ± SD	NAWM mean ± SD	WMH mean ± SD	Groups (hypertension vs. control)	ROIs (WMH vs. NAWM)
*Microglia (IBA1)*						
Frequency (#/mm^2^)^b^	270.1 ± 233.9	382.4 ± 236.7	278.4 ± 218.0	328.7 ± 194.9	*p* = *0.076*	*p* = *0.073*
Area (%)	2.1 ± 2.2	2.8 ± 2.2	2.2 ± 2.2	2.5 ± 1.9	*p* = 0.036 *	*p* = *0.129*
Intensity (%)	30.9 ± 1.5	34.2 ± 2.3	32.2 ± 2.2	35.4 ± 2.3	*p* = 0.010 *	*p* < 0.001 ***
Average length (µm)	24.8 ± 6.3	21.5 ± 5.4	22.2 ± 4.7	19.4 ± 3.58	*p* = *0.865*	*p* = 0.014 *
Circularity [0–1]	0.37 ± 0.06	0.44 ± 0.05	0.43 ± 0.05	0.50 ± 0.06	*p* = 0.002 **	*p* < 0.001 ***
*Astroglia (GFAP)*						
Area (%)	45.9 ± 17.1	69.1 ± 13.8	53.8 ± 27.3	69.7 ± 17.5	*p* = 0.005 **	*p* = 0.004 **
Intensity (%)	26.6 ± 1.9	32.9 ± 3.7	31.5 ± 7.9	36.4 ± 7.9	*p* < 0.001 ***	*p* = 0.012 *
*Perivascular inflammation*						
Perivascular inflammation present (%)	96.4 ± 8.1	100.0 ± 0.0	96.3 ± 5.5	99.8 ± 0.7	*p* = *0.943*	*p* = 0.030 *
Mild (%)	41.6 ± 36.9	6.5 ± 4.1	30.6 ± 28.7	7.5 ± 11.3	*p* = 0.017 *	*p* < 0.001***
Moderate (%)	50.8 ± 29.8	73.2 ± 13.1	51.8 ± 20.2	63.6 ± 16.2	*p* = *0.538*	*p* = 0.018 *
Severe (%)	7.6 ± 10.3	20.3 ± 11.4	17.6 ± 23.6	28.9 ± 20.4	*p* = 0.002 **	*p* = 0.002 **
Presence of close-range perivascular inflammation (%)	8.4 ± 11.6	16.1 ± 12.6	15.9 ± 17.5	31.7 ± 24.6	*p* = 0.006 **	*p* = 0.017 *

*GFAP* glial fibrillary acidic protein, *IBA1* ionized calcium-binding adaptor molecule 1, *NAWM* normal-appearing white matter, *ROIs* regions of interest, *SD* standard deviation, *WMH* white matter hyperintensity. *p < 0.05; **p < 0.01; ***p < 0.001

^a^ *p* values represent values after Bonferroni correction

^b^ Frequency describes the number of microglial cells per mm^2^

We found more microglial activation in *WMH* compared with NAWM in both groups (*p* < 0.001). Morphology-wise, microglial cells in WMH showed a reduced average length (shorter ramifications) (*p* = 0.014) and amoeboid shape (*p* < 0.001), indicating a that microglia within WMH of both individuals with and without hypertension show a phagocytic phenotype.

#### Astroglia

The astrogliotic area was significantly larger in individuals with *hypertension* compared to controls in both NAWM and WMH (*p* = 0.005; Fig. 2 and Table 2). Similarly, astrocytic activation was larger in individuals with hypertension (*p* < 0.001).

Astrocytic activation (*p* = 0.012) and area (*p* = 0.004) were overall higher in *WMH* compared to NAWM in both groups.

### Perivascular inflammation

**Fig. 3:**
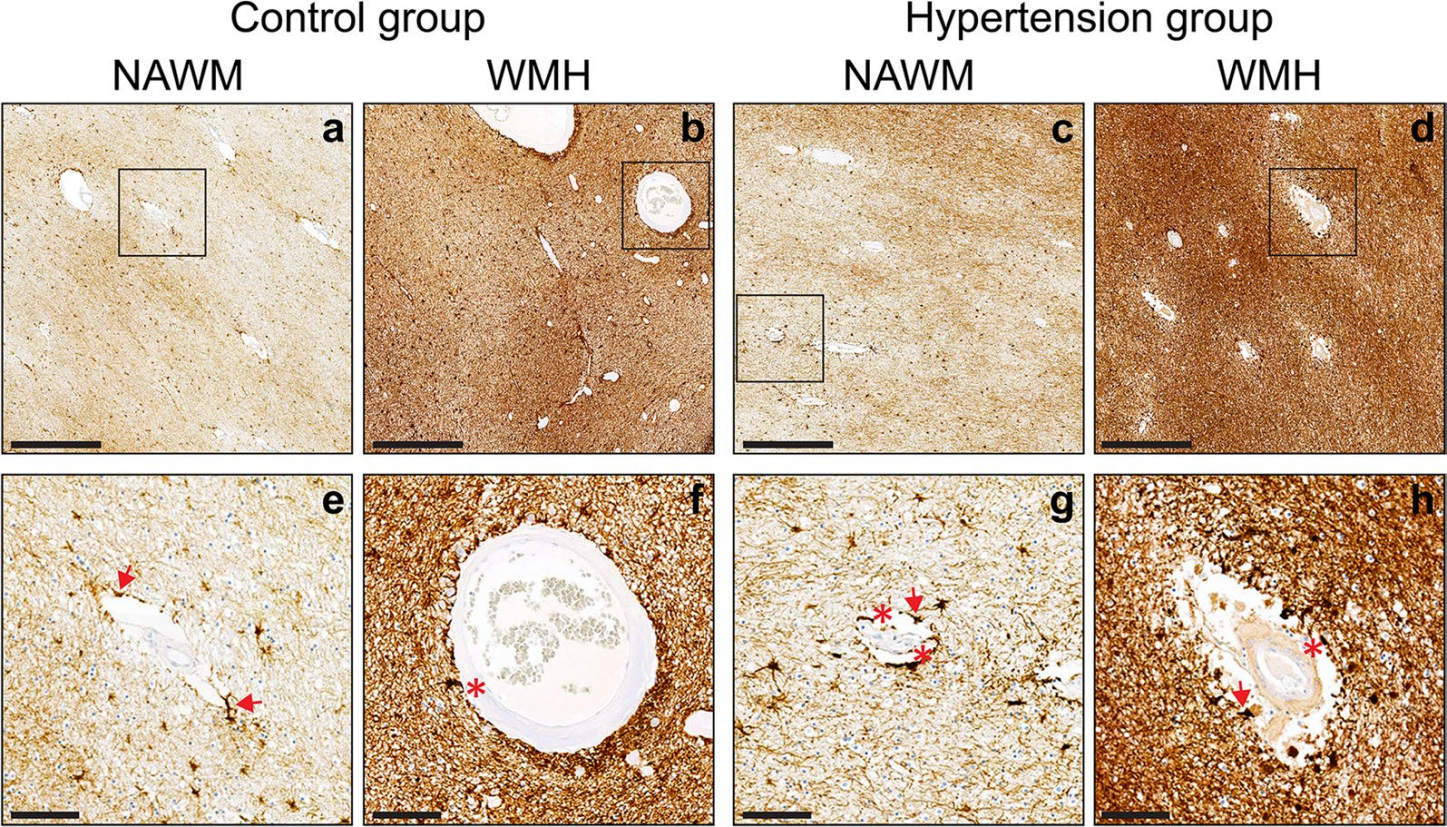
(Immuno-)histopathological characterization of perivascular inflammation of controls and individuals with hypertension within white matter. Representative images of perivascular inflammation assessed in glial fibrillary acidic protein (GFAP) stained sections at ×5 magnification of control individuals (**a**,** b**) and individuals with hypertension (**c**, **d**). **a**, **c** Correspond to normal-appearing white matter (NAWM) and **b**, **d** to white matter hyperintensity (WMH). The black boxes indicate regions of interest placed on **e**–**h** at ×20 magnification to visualize the (close-range) perivascular inflammation. The extent of perivascular inflammation (perivascular astroglia; red arrows) in individuals with hypertension (**c**, **d**) was more severe than controls (**a**, **b**), with a lower amount of vessels with signs of mild perivascular inflammation (*p* = 0.017) and more with a severe phenotype (*p* = 0.002). Individuals with hypertension (**g**, **h**) demonstrated more often close-range perivascular inflammation (red asterisks) than the control group (**e**, **f**) (*p* = 0.006). Perivascular inflammation was larger in WMH (**b**, **d**) compared to NAWM (**a**, **c**) for both groups (*p* = 0.030). WMH (**b**, **d**) showed less vessels with adjoining signs of mild inflammation (*p* < 0.001), and more vessels with severe perivascular inflammation (*p* = 0.002) than NAWM (**a**, **c**). Close-range perivascular inflammation was present twice as much in WMH (**b**, **d**) than NAWM (**a**, **c**) (*p* = 0.017). (Scale bar (**a**–**d**) = 500 µm; scale bar (**e**–**h**) = 100 µm)

In all individuals, signs of perivascular inflammation could be observed to some extent around the majority of blood vessels (> 95%). In comparison to controls, individuals with *hypertension* showed an overall larger amount of vessels with severe perivascular inflammation in both WMH and NAWM (*p* = 0.002; Fig. 3 and Table 2) and less vessels with mild perivascular inflammation (*p* = 0.017) compared to controls. Individuals with hypertension demonstrated more often close-range perivascular inflammation (24%) in both WMH and NAWM than the control group (12%; *p* = 0.006).

The extent of perivascular inflammation was larger in *WMH* compared to NAWM for both groups (*p* = 0.030), as almost all examined vessels within WMH (99.81%) showed some signs of perivascular inflammation. WMH showed ~ 80% less vessels with signs of mild perivascular inflammation (*p* < 0.001), while vessels showing severe perivascular inflammation were almost double (*p* = 0.002) compared with NAWM. Similarly, close-range perivascular inflammation was present twice as much in WMH (28%) than NAWM (14%; *p* = 0.017).

### Inflammation and the severity of WMH

Individuals with moderate to severe WMH burden showed more microglial activation (*p* = 0.007; Table 3) and larger microglia area (*p* = 0.047) than those with mild WMH burden; this difference was not found for astrogliosis or perivascular inflammation.

**Table 3:** Neurovascular inflammation by severity of WMH

	Mild burden mean ± SD	Moderate to severe burden mean ± SD	*p* value ^a^ Moderate to severe vs mild WMH
*Microglia (IBA1)*			
Frequency (#/mm^2^) ^*b*^	267.1 ± 175.6	364.8 ± 239.4	*p* = *0.145*
Area (%)	1.8 ± 1.5	3.1 ± 2.4	*p* = 0.047 *
Intensity (%)	32.9 ± 2.2	34.8 ± 2.9	*p* = 0.007 **
Average length (µm)	20.8 ± 4.6	21.9 ± 5.1	*p* = *0.065*
Circularity [0–1]	0.43 ± 0.06	0.46 ± 0.07	*p* = *0.352*
*Astroglia (GFAP)*			
Area (%)	61.4 ± 23.2	59.2 ± 22.6	*p* = *0.741*
Intensity (%)	33.6 ± 7.8	32.2 ± 7.5	*p* = *0.835*
*Perivascular inflammation*			
Perivascular inflammation present (%)	98.4 ± 4.8	97.6 ± 4.3	*p* = *0.591*
Mild (%)	19.6 ± 28.7	21.0 ± 22.4	*p* = *0.403*
Moderate (%)	59.8 ± 21.4	57.4 ± 19.1	*p* = *0.688*
Severe (%)	20.7 ± 18.2	21.7 ± 24.1	*p* = *0.736*
Presence of close-range perivascular inflammation (%)	20.4 ± 17.9	22.2 ± 24.7	*p* = *0.956*

*GFAP* glial fibrillary acidic protein,* IBA1* ionized calcium-binding adaptor molecule 1, *SD* standard deviation, *WMH* white matter hyperintensity. *p < 0.05; **p < 0.01

^a^ *p* values represent values after Bonferroni correction

^b^ Frequency describes the number of microglial cells per mm^2^

## Discussion

In this *post-mortem* (immuno-)histochemical verification of HF MRI markers of SVD we found higher neuro- and perivascular inflammation in individuals with hypertension, when compared to controls without hypertension. Further, in individuals with hypertension, neuroinflammation was not limited to the boundaries of MRI-visible lesions, but was also present in NAWM. In addition, we demonstrated that the extent of WMH burden was related to higher microglial activation, suggesting that microglial activation could be involved in the etiology of WMH. Taken together our data provide important new evidence that the occurrence of neuro- and perivascular inflammation at the tissue level within the CNS are key contributors to SVD pathogenesis in individuals with hypertension.

Neuroinflammation and vascular inflammation are increasingly recognized as risk factors for SVD [18]. Vascular and microstructural changes have been described in NAWM in previous studies, suggesting that pathological changes already occur in this peri-lesional tissue preceding conversion to WMH [29, 33]. However, whether neuro- and vascular inflammation was present in NAWM remained unknown. We observed that individuals with hypertension had a greater neuroinflammatory response, as illustrated by microglial activation and astrogliosis, and more severe perivascular inflammation compared to normotensives in both WMH and NAWM. This may suggest that hypertension is involved in both neuro- and vascular inflammation within the CNS. Furthermore, close-range perivascular inflammation was more often found in WMH and NAWM in individuals with hypertension compared to controls, suggesting that vascular inflammation might play a critical role in the pathogenesis of SVD in those with hypertension. As NAWM are areas at risk for WMH [33], increased neuro- and particularly perivascular inflammation within these regions could be an explanation for the observation that WMH burden was greater in those with hypertension. It is well known that hypertension promotes cerebrovascular atherosclerosis, impairing cerebral blood flow (CBF), and therewith white matter blood supply leading to ischemia [4, 11]. Vascular inflammation/endothelial disfunction within the brain might in turn exacerbate hypertension-induced endothelial damage and atherosclerosis in deep perforating arteries supplying the periventricular white matter [27]. This could then result in greater susceptibility to ischemic damage [16], and consequently worsen and/or accelerate WMH progression.

Neuroinflammation mediated by microglia and astroglia was higher in WMH compared to NAWM in both individuals with hypertension and controls. Microglia are central in neuroinflammation [24]. Microglial activation is known to correlate with IBA1 upregulation [10]. However, important changes are also linked to microglia activation as these show de-ramification and rounder/more ameboid morphology and phagocytic function [28]. Here, we observed that microglial activation in WMH was not solely mediated by IBA1 upregulation, but also that amoeboid phagocytic microglia were present at large. Phagocytic microglia can target different substrates, including myelin debris [7]. While under normal conditions this can be beneficial to maintain homeostasis, it is believed that with aging phagocytosis becomes impaired, eventually promoting more neuroinflammation and neurodegeneration [7]. Thus, phagocytic microglia in aged individuals might play an important role in WMH pathogenesis as these could be responsible for the myelin loss that is thought to underlie these lesions.

Finally, we examined both neuro- and perivascular inflammation across WMH burden to unveil WMH etiology. We observed larger microglial activation in moderate to severe WMH burden compared to mild WMH burden, suggestion of a causal role. Conversely, we did not observe changes in astrogliosis nor in perivascular inflammation. Thus, moderate to severe WMH burden showed an overall greater IBA1 upregulation, but not yet increased phagocytic microglia. This suggests that microglial activation is present in both individuals with and without hypertension with moderate to severe WMH burden. Given that the presence of phagocytic microglia was not greater in individuals with moderate to severe WMH burden and that these may account for myelin loss, we suggest that anti-inflammatory therapies, which may prevent conversion to their phagocytic phenotype, might prove beneficial in ameliorating WMH progression particularly in those with moderate WMH with a high risk of developing severe WMH [3]. Second, perivascular inflammation is not directly associated with increased WMH burden. This is in line with the fact that we did not find any correlation across neurovascular inflammation and WMH volume (data not shown; see Additional file 2). Rather, perivascular inflammation is strongly associated with hypertension.

This study has several important clinical implications. To this date, many therapeutical approaches for SVD have focused on antihypertensive treatment (reviewed by [31]). While most studies have shown inconsistent results (reviewed by [31]), the SPRINT MIND clinical trial recently showed that intensive blood lowering treatment (< = 130 mm Hg) leads to a small reduction of WMH progression compared to standard blood pressure lowering treatments [22]. Searching for more beneficial alternatives, treatment of inflammation in individuals with hypertension has recently drawn considerable interest. Anti-inflammatory drugs, e.g., colchicine, are currently being investigated as add-on to secondary prevention after mild-to-moderate ischemic stroke (CONVINCE clinical trial [13]). Our findings showed that perivascular inflammation in WMH and NAWM was twice as common in patients with hypertension, confirming the strong link between hypertension and vascular inflammation in the CNS. Our findings may pave the road for future clinical studies to investigate the role of anti-inflammatory drugs on WMH progression. Second, as higher WMH burden correlated with greater microglial activation, patients showing WMH might benefit from novel personalized, rather than one-size-fits-all therapeutic approaches in the context of SVD. For instance, preclinical studies on microglia depletion showed beneficial effects of lowering neuroinflammatory responses in the context of hypertension and SVD [14]. Therefore, future studies should investigate novel multitarget interventions targeting both hypertension and neuro(vascular)inflammation.

The present study provides the first comprehensive assessment of both neuro- and perivascular inflammation within WMH and NAWM in individuals with and without hypertension. Yet, the limitations of this study include its cross-sectional nature, which is inevitable in *post-mortem* (immuno-)histopathological analysis, but hinders the understanding of the multifactorial pathological cascade resulting in WMH. Although there are several important SVD hallmarks according to the STRIVE criteria [32], we solely focused on changes between WMH and NAWM. Therefore, next steps will include the assessment of the role of neurovascular inflammation and blood–brain barrier (BBB) integrity, as well as the ever increasing spectrum of (novel) markers of SVD such as enlarged perivascular spaces, microbleeds, but also for example (acute) microinfarcts. Furthermore, vascular inflammation was only assessed through studying the extent of perivascular astrogliosis, and future studies including specific markers for vascular inflammation such as immune cells (infiltration), CD68 (expressed by monocytic phagocytes), matrix metallopeptidase 9 (MMP-9) are needed to further elucidate the relationship between vascular inflammation and/or BBB breakdown in SVD.

This work has several strengths. This is the first study investigating both neuro- and perivascular inflammation underlying WMH in the context of sporadic SVD using HF MRI in individuals who had hypertension and age-matched controls. Highlighting the crucial role of neuroinflammation in SVD, particularly of vascular inflammation in individuals with hypertension. Secondly, we carefully designed a custom software-based registration approach where several anatomical landmarks were considered to register MRI-pathology, which later allowed us to use MRI-segmented WMH and NAWM to accurately analyze both neuro- and perivascular inflammation underlying these regions.

## Conclusion

In conclusion, our findings of increased activated microglia, astrogliosis and perivascular neuroinflammation in WMH and, to a lesser extent, in areas at risk of developing into WMH, i.e., NAWM, in individuals with hypertension, imply that both neuro- and vascular inflammation play crucial roles in the pathogenesis of WMH. Our findings suggest that neurovascular inflammation is a key mechanism of (NAWM conversion to) WMH in individuals with hypertension. Therefore, future treatment strategies may include multi-domain interventions, including anti-inflammatory treatment targeting neurovascular inflammation and hypertension, to further understand and prevent the progression of WMH.

## Supplementary Information

**Additional file 1: Table S1**. Detailed case characteristics (hypertension, gender, age and cause of death). Table S1 includes detailed case specific characteristics (hypertension, gender, age, year of death and cause of death). Causes of death were extracted from final pathology reports.

**Additional file 2: Table S2.** Pearson correlation between WMH volume and neurovascular inflammation. Table S2 shows the Pearson correlation (r) scores as well as the *p values* after Bonferroni correction.

## Data Availability

The datasets during and/or analyzed during the current study available from the corresponding author on reasonable request.

## Abbreviations

A: Anterior
ANOVA: Analysis of variance
BBB: Blood–brain barrier
BMI: Body mass index
CBF: Cerebral blood flow.
CNS: Central nervous system
DAB: 3,3'-Diaminobenzidine
FLAIR: Fluid-attenuated inversion recovery
GFAP: Glial fibrillary acidic protein
HE: Haematoxylin/eosin
HF: High field
IBA1: Ionized calcium-binding adaptor molecule 1
L: Left
MANGO: Multi-image Analysis GUI
MMP-9: Matrix metallopeptidase 9
MRI: Magnetic resonance imaging
NAWM: Normal-appearing white matter
P: Posterior
PET: Positron emission tomography
R: Right
ROIs: Regions of interest
SD: Standard deviation
SVD: Small vessel disease
TE: Echo time
TIFF: Tag image file format
TR: Repetition time
WMH: White matter hyperintensity

## References

[1] Abraham HM, Wolfson L, Moscufo N, Guttmann CR, Kaplan RF, White WB (2016). Cardiovascular risk factors and small vessel disease of the brain: Blood pressure, white matter lesions, and functional decline in older persons. J Cereb Blood Flow Metab.

[2] Anttila JE, Whitaker KW, Wires ES, Harvey BK, Airavaara M (2017). Role of microglia in ischemic focal stroke and recovery: focus on Toll-like receptors. Prog Neuropsychopharmacol Biol Psychiatry.

[3] Cai M, Jacob MA, Loenen MRV, Bergkamp M, Marques J, Norris DG, Duering M, Tuladhar AM, Leeuw F-ED (2022). Determinants and temporal dynamics of cerebral small vessel disease: 14-year follow-up. Stroke.

[4] Cannistraro RJ, Badi M, Eidelman BH, Dickson DW, Middlebrooks EH, Meschia JF (2019). CNS small vessel disease. Neurology.

[5] Evans LE, Taylor JL, Smith CJ, Pritchard HAT, Greenstein AS, Allan SM (2021). Cardiovascular comorbidities, inflammation, and cerebral small vessel disease. Cardiovasc Res.

[6] Fazekas F, Chawluk JB, Alavi A, Hurtig HI, Zimmerman RA (1987). MR signal abnormalities at 1.5 T in Alzheimer's dementia and normal aging. Am J Roentgenol.

[7] Galloway DA, Phillips AEM, Owen DRJ, Moore CS (2019). Phagocytosis in the brain: homeostasis and disease. Front Immunol.

[8] Hilal S, Mok V, Youn YC, Wong A, Ikram MK, Chen CL (2017). Prevalence, risk factors and consequences of cerebral small vessel diseases: data from three Asian countries. J Neurol Neurosurg Psychiatry.

[9] Hiner MC, Rueden CT, Eliceiri KW (2017). ImageJ-MATLAB: a bidirectional framework for scientific image analysis interoperability. Bioinformatics.

[10] Hopperton KE, Mohammad D, Trépanier MO, Giuliano V, Bazinet RP (2018). Markers of microglia in post-mortem brain samples from patients with Alzheimer's disease: a systematic review. Mol Psychiatry.

[11] Iadecola C, Park L, Capone C (2009). Threats to the mind: aging, amyloid, and hypertension. Stroke.

[12] Jurga AM, Paleczna M, Kadluczka J, Kuter KZ (2021). Beyond the GFAP-astrocyte protein markers in the brain. Biomolecules.

[13] Kelly P, Weimar C, Lemmens R, Murphy S, Purroy F, Arsovska A, Bornstein NM, Czlonkowska A, Fischer U, Fonseca AC (2021). Colchicine for prevention of vascular inflammation in Non-CardioEmbolic stroke (CONVINCE)—study protocol for a randomised controlled trial. Eur Stroke J.

[14] Kerkhofs D, van Hagen BT, Milanova IV, Schell KJ, van Essen H, Wijnands E, Goossens P, Blankesteijn WM, Unger T, Prickaerts J (2020). Pharmacological depletion of microglia and perivascular macrophages prevents Vascular Cognitive Impairment in Ang II-induced hypertension. Theranostics.

[15] Lancaster JL, Martinez MJ (2016) Mango (Multi-image Analysis GUI). Multi-image Analysis GUI, City

[16] Lin J, Wang D, Lan L, Fan Y (2017). Multiple factors involved in the pathogenesis of white matter lesions. Biomed Res Int.

[17] Low A, Mak E, Malpetti M, Passamonti L, Nicastro N, Stefaniak JD, Savulich G, Chouliaras L, Su L, Rowe JB (2020). In vivo neuroinflammation and cerebral small vessel disease in mild cognitive impairment and Alzheimer's disease. J Neurol Neurosurg Psychiatry.

[18] Low A, Mak E, Rowe JB, Markus HS, O'Brien JT (2019). Inflammation and cerebral small vessel disease: a systematic review. Ageing Res Rev.

[19] MathWorks (2020) Approaches to Registering Images—MATLAB & Simulink. https://nl.mathworks.com/help/images/approaches-to-registering-images.html. Accessed September 09 2020

[20] MathWorks (2021) Image Registration—MATLAB & Simulink. https://www.mathworks.com/help/images/image-registration.html?s_tid=CRUX_lftnav. Accessed May 17 2021

[21] McGeer EG, McGeer PL (2001). Innate Immunity in Alzheimer's Disease: a model for local inflammatory reactions. Mol Interv.

[22] Nasrallah IM, Pajewski NM, Auchus AP, Chelune G, Cheung AK, Cleveland ML, Coker LH, Crowe MG, Cushman WC, Cutler JA (2019). Association of intensive vs standard blood pressure control with cerebral white matter lesions. JAMA.

[23] Niemisto A, Dunmire V, Yli-Harja O, Zhang W, Shmulevich I (2005). Robust quantification of in vitro angiogenesis through image analysis. IEEE Trans Med Imaging.

[24] Nimmerjahn A, Kirchhoff F, Helmchen F (2005). Resting microglial cells are highly dynamic surveillants of brain parenchyma in vivo. Science.

[25] Ruifrok AC, Johnston DA (2001). Quantification of histochemical staining by color deconvolution. Anal Quant Cytol Histol.

[26] Shi Y, Wardlaw JM (2016). Update on cerebral small vessel disease: a dynamic whole-brain disease. Stroke Vasc Neurol.

[27] Szmitko PE, Wang CH, Weisel RD, Jeffries GA, Anderson TJ, Verma S (2003). Biomarkers of vascular disease linking inflammation to endothelial activation: part II. Circulation.

[28] Vainchtein ID, Molofsky AV (2020). Astrocytes and microglia: in sickness and in health. Trends Neurosci.

[29] van Leijsen EMC, Bergkamp MI, van Uden IWM, Ghafoorian M, van der Holst HM, Norris DG, Platel B, Tuladhar AM, de Leeuw FE (2018). Progression of white matter hyperintensities preceded by heterogeneous decline of microstructural integrity. Stroke.

[30] Wardlaw JM, Smith C, Dichgans M (2013). Mechanisms of sporadic cerebral small vessel disease: insights from neuroimaging. Lancet Neurol.

[31] Wardlaw JM, Smith C, Dichgans M (2019). Small vessel disease: mechanisms and clinical implications. Lancet Neurol.

[32] Wardlaw JM, Smith EE, Biessels GJ, Cordonnier C, Fazekas F, Frayne R, Lindley RI, O'Brien JT, Barkhof F, Benavente OR (2013). Neuroimaging standards for research into small vessel disease and its contribution to ageing and neurodegeneration. Lancet Neurol.

[33] Wardlaw JM, Valdes Hernandez MC, Munoz-Maniega S (2015). What are white matter hyperintensities made of? Relevance to vascular cognitive impairment. J Am Heart Assoc.

[34] Yushkevich PA, Piven J, Hazlett HC, Smith RG, Ho S, Gee JC, Gerig G (2006). User-guided 3D active contour segmentation of anatomical structures: significantly improved efficiency and reliability. Neuroimage.

